# Predictive factors of mortality in patients with pelvic fracture and shock submitted to extraperitoneal pelvic packing

**DOI:** 10.1590/0100-6991e-20223259-en

**Published:** 2022-09-21

**Authors:** VINICIUS CORDEIRO FONSECA, CARLOS AUGUSTO METIDIERI MENEGOZZO, JULIANA MYNSSEN DA FONSECA CARDOSO, CELSO OLIVEIRA BERNINI, EDIVALDO MASSAZO UTIYAMA, RENATO SÉRGIO POGGETTI

**Affiliations:** 1 - Hospital das Clínicas da Faculdade de Medicina da USP, Departamento de Cirurgia de Emergência, Divisão de Cirurgia Geral e Trauma - São Paulo - SP - Brasil

**Keywords:** Shock, Hemorrhage, Pelvic Bones, Multiple Trauma, Mortality, Hemorragia, Fraturas do Quadril, Ossos Pélvicos, Ferimentos e Lesões, Tampões de Gaze Cirúrgicos

## Abstract

**Introduction:**

in recent decades, the extraperitoneal pelvic packing technique has been disseminated, but there are still few studies. Thus, it was decided to analyze the results of extraperitoneal pelvic tamponade, in patients with pelvic fracture and shock, in order to identify predictive factors for mortality.

**Methods:**

a retrospective review of medical records of patients submitted to extraperitoneal pelvic packing was conduced. We analyzed their characteristics, prehospital and emergency room data, pelvic fracture classification, associated and severity injuries, laboratory and imaging exams, data on packing, arteriography, and other procedures performed, complications, hemodynamic parameters, and amount of transfused blood products before and after packing.

**Results:**

data were analyzed from 51 patients, who showed signs of shock from prehospital care, presence of acidosis, with high base deficit and arterial lactate levels. Most patients underwent multiple surgical procedures due to severe associated injuries. The incidence of coagulopathy was 70.58%, and overall mortality was 56.86%. The group of non-surviving patients presented significantly higher age, prehospital endotracheal intubation, and lower Glasgow Coma Scale scores (p<0.05). The same group presented, before and after extraperitoneal pelvic packing, significantly worse hemodynamic parameters of mean arterial pressure, pH, base deficit, hemoglobin, and arterial lactate (p<0.05). The non-surviving group received significantly more units of packed red blood cells, fresh frozen plasma and platelets within 24 hours following extraperitoneal pelvic packing (p<0.05).

**Conclusion:**

age and base deficit are independent predictors of mortality in patients submitted to extraperitoneal pelvic packing.

## RESUMO

**Introdução:**

nas últimas décadas, tem sido difundida a técnica de tamponamento pélvico extraperitoneal, porém ainda existem poucos estudos. Decidiu-se analisar os resultados do tamponamento extraperitoneal de pelve, em pacientes com fratura pélvica e choque, com objetivo de identificar fatores preditivos de mortalidade.

**Métodos:**

foi realizada revisão do prontuário dos pacientes submetidos ao tamponamento extraperitoneal de pelve. Foram analisadas as características dos pacientes, dados do atendimento pré-hospitalar e na sala de emergência, classificação da fratura, presença de lesões associadas, exames laboratoriais e de imagem, dados relativos ao tamponamento, e outros procedimentos realizados, complicações, parâmetros hemodinâmicos e quantidade de hemoderivados transfudidos.

**Resultados:**

foram analisados os dados de 51 pacientes, com sinais de choque desde o atendimento pré-hospitalar, presença de acidose, elevado déficit de bases e lactato arterial. Houve alta prevalência de lesões graves associadas, requerendo múltiplos procedimentos cirúrgicos. A incidência de coagulopatia foi 70,58% e mortalidade 56,86%. O grupo de pacientes não sobreviventes apresentou idade e intubação orotraqueal pré-hospitalar maiores, e escores na escala de coma de Glasgow menores (p<0,05). O mesmo grupo apresentou, antes e após o tamponamento extraperitoneal de pelve, parâmetros hemodinâmicos menores de pressão arterial média, pH, déficit de bases e hemoglobina, e maior de lactato arterial (p<0,05). O grupo de pacientes não sobreviventes recebeu mais concentrados de hemácias, plasma fresco congelado e concentrado de plaquetas nas 24h seguintes ao tamponamento extraperitoneal de pelve (p<0,05).

**Conclusão:**

idade e o excesso de bases são fatores preditivos independentes de mortalidade em pacientes submetidos ao tamponamento extraperitoneal de pelve.

## INTRODUCTION

Hemorrhage is the most common cause of potentially preventable death in trauma patients, and pelvic fractures are among the main injuries in this group^1^. Mortality in patients with pelvic fractures and shock ranges from 21% to 66%^2^ ^-^ ^4^. In the last two decades, extraperitoneal pelvic packing (EPP) has been indicated in patients with hemodynamic instability refractory to initial fluid resuscitation, acquiring a role as a priority procedure in damage control in multidisciplinary protocols^5^ ^-^ ^16^. Even so, there is still no consensus on the best sequence of procedures in the treatment of these patients^17^, and despite the advantages described, EPP has not been routinely used. In a recent survey of directors of North American level-I trauma centers, only 30% considered it effective and none prioritized EPP over arteriography^18^. Another recent US multicenter study showed that EPP was performed in only 5.61% of such patients^19^.

There are still few studies reporting EPP results. In addition, the validity of their results may not extrapolate to the reality of hospitals in developing countries, where trauma systems do not exist and more sophisticated resources, such as arteriography, are limited or unavailable.

The objective of this study is to identify predictive factors of mortality in patients with pelvic fracture and hemodynamic instability undergoing EPP in a Brazilian referral hospital for trauma care.

## METHODS

We carried out a retrospective review of medical records and laboratory and imaging tests. We included trauma patients with pelvic fracture and shock who underwent EPP from October 2010 to December 2016. We excluded individuals presenting with cardiopulmonary arrest during prehospital care or at arrival at the Emergency Room (ER), or who died in the operating room before the end of the EPP.

The variables obtained from medical records were epidemiological characteristics, associated injuries, prehospital endotracheal intubation, and transport time. At admission, data were collected on hemodynamic parameters, Glasgow Coma Scale, laboratory tests, use of tranexamic acid, temporary immobilization of the pelvis, classification of pelvic fracture, FAST ultrasound result, and presence of arterial contrast extravasation on CT scan (TC). We also collected intraoperative data related to EPP, external fixation (EF) of the pelvis, exploratory laparotomy, arteriography, and other procedures, as well as the amount of packed red blood cells (PRBC), fresh frozen plasma (FFP), and platelets units transfused from admission to 24 hours after EPP. In addition, we gathered data on the diagnosis of coagulopathy up to 12 hours after EPP, hemodynamic and laboratory parameters before and after EPP, and mortality.

We defined shock as Class III or IV hemorrhage, according to ATLS^20^. We classified all injuries according to the 2005 AIS (Abbreviated Injury Score - 2008 update) scale. Subsequently, we calculated the Injury Severity Score (ISS) and the New Injury Severity Score (NISS) for each patient. We considered an associated extrapelvic injury a cause of shock based on the report of massive active bleeding during the initial assessment or in the operating room, or the presence of non-hemorrhagic shock (neurogenic shock or tension pneumothorax). We defined the type of pelvic fracture using the Young & Burgess classification. EPP was performed in patients with pelvic fracture and shock who did not have a sustained hemodynamic response after initial resuscitation with 2,000ml of crystalloid solution. This management relied on the ATLS recommendations at the time of the study.

We considered coagulopathy the presence of at least one criterion: clinical, laboratory, or thromboelastometry (ROTEM^®^), when performed. Conventional laboratory tests with a result 1.5 times greater than the reference value of any of the following tests, such as prothrombin time (PT), international normalized ratio (INR), activated partial thromboplastin time (APTT), serum fibrinogen <100mg/dl or quantitative platelet count <100,000/ml were deemed as coagulopathy. For the analysis of mortality, we considered the period of up to 30 days after EPP.

Arteriography indications were arterial contrast extravasation on computed tomography (CT) or refractory shock after operative approaches (EPP and external fixation, when performed).

We divided the included patients into two groups: survivors and non-survivors. To compare the hemodynamic parameters between the two groups, we considered three phases: before the EPP (operating room, immediately before the procedure), 3h after EPP, and 6h after EPP. For the comparison of blood transfusions, we considered two phases: from admission to EPP and 24 hours after EPP.

We summarized qualitative variables in absolute and relative frequencies and compared them with the chi-square test, Fisher’s exact test, or likelihood ratio. We summarized quantitative variables as mean, standard deviation, median, minimum, and maximum, and compared them using the Student’s t or Mann-Whitney tests. For univariate analysis, we compared variables between survivors and non-survivors. The multivariate analysis model consisted of variables that were related to death at a significance level of up to 0.20, and that we considered clinically relevant by the stepwise method. For all comparisons, we used a significance level of 0.05 to reject the null hypothesis. The software used was the SPSS for Windows, version 19.0.

## RESULTS

**Figure 1:**
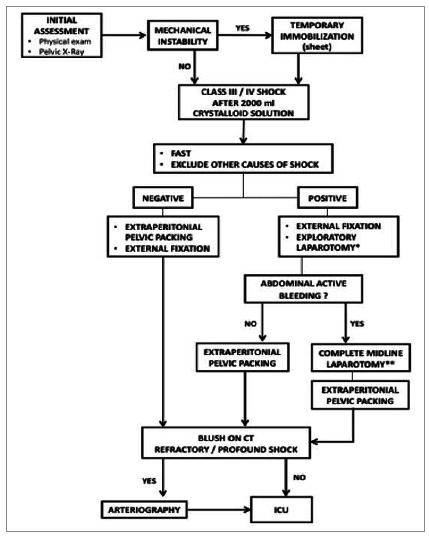
Algorithm for evaluation and treatment of unstable pelvic fractures. *Median incision from the xiphoid process to the umbilicus, keeping it separate from the extraperitoneal pelvic tamponade incision. **Extended laparotomy incision below the umbilicus, keeping the pelvic peritoneum intact.

In the analyzed period, 58 patients met the inclusion criteria, of whom we excluded seven, the sample analyzed in the study then consisting of 51 individuals. All patients were victims of high-energy blunt trauma, and the mortality rate was 56.86%. Figure 1 shows the algorithm for assessment and treatment of unstable pelvic fractures.

Table 1 shows the comparison between the groups of survivors and non-survivors regarding epidemiological characteristics, associated injuries, prehospital, and emergency room data.

**Table 1:** Comparison of epidemiological characteristics, prehospital, and emergency room data between survivors and non-survivors.

	Survivors	Non-survivors	p
Age years	26.8 ±10.8	34.7 ±14.9	0.041
Male, n (%)	16 (72.72)	22 (75.86)	1
Associated injuries*, n (%)	22 (100)	27 (93.1)	
ISS (Injury Severity Score)	38 [29-66]	41 [25-75]	0.142
NISS (New Injury Severity Score)	43 [32-66]	50 [25-75]	0.088
Extrapelvic injury justifying shock, n (%)	17 (77.3)	22 (75.9)	1
Prehospital transport time, minutes	38.2 (±16.5)	50.3 (±50.8)	0.454
	Survivors	Non-survivors	p
Prehospital intubation, n (%)	4 (26.7)	15 (68.2)	0.032
HR, bpm	133.9 (±16)	121 (±29)	0.054
SBP, mmHg	91.2 (±34.5)	77.6 (±37.6)	0.192
Glasgow Coma Scale, points	14 [3-15]	6.5 [3-15]	0.049
pH	7.2 (±0.1)	7.2 (±0.1)	0.418
Base deficit, mmol/l	-10.5 (±3.3)	-12.6 (±6.9)	0.436
Arterial lactate, mg/dl*	39.6 (±32.6)	62.1 (±33.5)	0.16
Hb, mg/dl	9.8 (2)	9.6 (2.6)	0.852
Tranexamic acid <3h, n (%)	16 (72.7)	20 (69)	0.768
Temporary immobilization, n (%)	17 (81)	20 (71.4)	0.666
patients, n	22	29	

*Reference values in the HC-FMUSP laboratory: 4.5 to 14.4mg/dl; bpm: beats per minute; Hb: hemoglobin; HR: heart rate; n: number of patients; SBP: systolic blood pressure.

**Figure 2:**
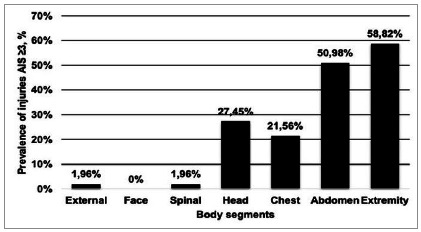
Prevalence of severe injury (AIS ≥3) according to body segment

Forty-nine patients (96.07%) had associated injuries and 76.47% had at least one extrapelvic injury that could justify shock, but there was no significant difference between the groups. Figure 2 and Table 2 show the distribution of extrapelvic injuries by body segment and those that could justify shock, respectively. The time elapsed between the trauma and the patient’s admission to the emergency department was 45.4 ± 40.4 minutes. On admission to the emergency room, patients were tachycardic and hypotensive. Eight patients had no measurable systolic blood pressure (SBP). Only one individual had a heart rate (HR) below 100 bpm and SBP above 90mmHg. In the initial evaluation, 75.5% of the patients had pelvic immobilization with a sheet and 70.58% received 1g of tranexamic acid in the first three hours after trauma.

**Table 2:** Prevalence of extrapelvic injury that justifies shock.

	Total
Neurogenic shock, n (%)	1 (1.96)
External bleeding, n (%)	2 (3.92)
Chest injury, n (%)	7 (13.72)
Abdominal injury, n (%)	13 (25.49)
Extremity injury (limbs), n (%)	29 (56.86)

n: number of patients.

Three patients in the non-survivor group did not undergo any imaging exams and the pelvic fractures could not be classified. All showed signs of shock during prehospital care, severe associated injuries, coagulopathy, underwent exploratory laparotomy, and died within the first 24 hours of hospitalization. In these patients, physical examination revealed an unstable pelvic fracture. There was no significant difference between the types of fracture according to the Young & Burgess classification between survivors and non-survivors (likelihood ratio, p=0.504). One patient in each group had a type I lateral compression fracture. Among them, one had retropubic arterial bleeding on CT.

Forty-seven patients underwent FAST, with a positive result in 48.9%, there being no significant difference between groups [10 survivors (50%) vs. 19 non-survivors (70.4%), p=0.264]. Six of these patients (12.76%) had false positive results as no intraperitoneal blood was found during laparotomy (two patients in the survivor group and four patients in the non-survivor group). Of the four patients who did not undergo FAST, one was referred directly to the operating room upon arrival, and one had traumatic evisceration. For the remaining two cases, ultrasound equipment was not available at the time.

Sixteen patients (31.37%), eight from each group, underwent CT during initial care before EPP. There was no significant difference in the incidence of arterial contrast extravasation between groups [three survivors (37.5%) vs. five non-survivors (62.5%), p=0.619], and CT was not associated with mortality [eight survivors (36.36%) vs. eight non-survivors (27.58%), p=504].

Table 3 shows the comparison of data related to EPP, external fixation, and pelvic arteriography between survivors and non-survivors.

**Table 3:** Comparison of data related to extraperitoneal pelvic tamponade, external fixation, and arteriography between the groups of survivors and non-survivors.

	Survivors	Non-survivors	p
Extraperitoneal tamponade			
Time between admission and EPP, min	144 (±118.8)	131 (±82.6)	0.64
Surgical packs, n	8 [3-14]	8 [4-12]	0.528
Patients, n	22	29	
External fixation			
Patients undergoing EF, n (%)	19 (86.36)	23 (79.31)	0.713
Patients, n	22	29	
Time between EPP and EF, min	20 [5-305]	20 [5-120]	0.47
Patients, n	17	20	
Arteriography			
AR indication, n (%)	16 (72.72)	25 (86.2)	0.286
Arterial contrast extravasation on CT, n (%)	3 (13.63)	5 (17.24)	1
Refractory shock^†^, n (%)	13 (59.09)	20 (68.96)	0.465
Patients, n	22	29	
Arteriography performed	10 (45.45)	10 (34.48)	0.61
Patients, n	22	29	
Time between EPP and AR, min	195 [90-1185]	150 [40-860]	0.693
Patients, n	10	10	
Presence of arterial injury	3 (30)	5 (50)	0.65
Embolization	4 (40)	4 (50)	1
Patients, n	10	10	

^†^: refractory shock after EPP (and external fixation of the pelvis, when performed); AR: arteriography; CT: computed tomography; EF: external fixation of the pelvis; EPP: extraperitoneal pelvis tamponade; n: number of patients.

Thirty-one patients (60.78%) underwent exploratory laparotomy before EPP. There was no significant difference between groups regarding the indication of exploratory laparotomy [13 survivors (59.1%) vs. 18 non-survivors (62.1%), p=1], presence of abdominal injury [nine survivors (69.23%) vs. 11 non-survivors (61.11%), p=0.718], or prevalence of non-therapeutic laparotomy [seven survivors (53.84%) vs. eight non-survivors (44.44%), p=0.879]. Other surgical procedures were performed by other specialties in addition to EPP and EF in 82.35% of patients [19 survivors (86.36%) vs. 23 non-survivors (79.31%), p=0.714]. There was no difference in the total time between admission and the end of all surgical procedures between groups [454 minutes (±273.1) for survivors vs. 380.2 minutes (±153.3) for non-survivors, p=0.276].

No patient underwent arteriography before EPP. After EPP and EF, 41 patients (80.39%) had an indication for arteriography. Among these, the procedure was performed in 20 patients (48.78%). Of the 21 patients with arteriography indication who did not undergo the procedure, 15 died.

Table 4 shows the comparison of hemodynamic parameters and the amount of blood products transfused between the surviving and non-surviving patients. The incidence of coagulopathy within 12 hours after EPP was 70.58%, with no significant difference between groups [15 survivors (68.18%) vs. 21 non-survivors (72.41%), p=0.985].

**Table 4:** Comparison of hemodynamic parameters and transfusion of blood products between survivors and non-survivors before and after extraperitoneal pelvis packing.

	Survivors	Non-survivors	p	NVR
Heart rate				
Pre-EPP, bpm	125 (±19)	126.4 (±23.7)	0.824	51
3h post-EPP, bpm	114.8 (±20.4)	121.2 (±24.8)	0.328	51
6h post-EPP, bpm	115.9 (±21.1)	118 (±23.9)	0.753	45
Mean blood pressure				
Pre-EPP, mmHg	67.7 (±18.8)	45.4 (±20.2)	<0.001	51
3h post-EPP, mmHg	74.5 (±9.4)	61 (±18.2)	0.001	51
6h post-EPP, mmHg	76.5 (±8.9)	65.6 (±18.8)	0.017	45
pH				
Pre-EPP	7.2 (±0.1)	7.1 (±0.2)	0.001	48
3h post-EPP	7.28 (±0.06)	7.17 (±0.11)	<0.001	47
6h post-EPP	7.3 (±0.06)	7.19 (±0.11)	<0.001	42
Base deficit				
Pre-EPP, mmol/L	-10.79 (3.99)	-14.55 (6.13)	0.015	47
3h post-EPP, mmol/L	-6.5 (2.7)	-10.4 (4.4)	0.001	47
6h post-EPP, mmol/L	-5.6 (3.2)	-8.9 (5.6)	0.028	41
Arterial lactate				
Pre-EPP, mg/dl	36.5 [8-103]	92.5 [12-190]	<0.001	48
3h post-EPP, mg/dl	50 [17-98]	113 [15-243]	<0.001	48
6h post-EPP, mg/dl	55.3 (±36.1)	115.4 (±76.8)	0.003	41
Hemoglobin				
Pre-EPP, mg/dl	9.65 [5.8-16]	8 [4.1-13.3]	0.013	50
3h post-EPP, mg/dl	10.2 (±1.7)	8 (±2.6)	0.001	48
6h post-EPP, mg/dl	10.2 (±1.3)	8.9 (±2.8)	0.054	41
Packed red blood cells (PRBC)				
Pre-EPP, units	2.3 (±2.1)	2.3 (±1.4)	0.94	51
24h post-EPP, units	5.1 (±3.2)	8.5 (±5.6)	0.01	51
Fresh frozen plasma (FFP)				
Pre-EPP, units	0.7 (±1.4)	1 (±1.6)	0.575	51
24h post-EPP, units	3.2 (±3.6)	7.8 (±7.4)	0.006	51
Platelets (PLQ)				
Pre-EPP, units	0.1 (±0.6)	0.3 (±1.1)	0.425	51
24h post-EPP, units	4.5 (±3.9)	7.5 (±5.7)	0.042	51
Total patients	22	29		

HR: heart rate; PAM; mean arterial pressure; DB: base deficit; Hb: hemoglobin; PRBC: packed red blood cells; FFP: fresh frozen plasma; PLQ: platelets; EPP: extraperitoneal pelvis tamponade; NVR: number of valid observations; p: significance level.

### Multivariate analysis

The variables selected by statistical criteria and clinical importance were age, 24-hour red blood cell transfusion after EPP, NISS, and base deficit before EPP. Table 5 describes the multivariate analysis model used. Age (OR 1.07 [1.01-1.14], p=0.022) and base deficit before EPP (OR 0.81 [0.68-0.97], p=0.022) were identified as independent predictors of mortality.

**Table 5:** Multivariate logistic regression model to predict death (Yes x No), n=47).

Variables	Estimation	OR [CI (95%)]	p
Age	0.069	1.07 (1.01-1.14)	0.022
Pre-EPP transfused PRBC	0.162	1.18 (0.98-1.41)	0.075
NISS	0.060	1.06 (0.999-1.13)	0.052
Pre-EPP BD	-0.205	0.81 (0.68-0.97)	0.022

BD: base deficit; CI: confidence interval; EPP: extraperitoneal pelvis tamponade; n: number of patients; NISS: New Injury Severity Score; OR (Odds ratio): odds ratio; p: significance level; PRBC: packed red blood cells.

## DISCUSSION

This study evaluated predictive factors of mortality in patients with pelvic fracture and hemodynamic instability undergoing extraperitoneal pelvic packing. After multivariate analysis, older age and base deficit before EPP were identified as independent predictors of mortality. Older age was associated with mortality in other series that studied EPP^14^ ^,^ ^21^. Older patients tend to have a worse prognosis, even in mechanically stable fractures^22^. Correa et al.^23^ reported that a risk of death in patients older than 50 years 28.3 times higher than in patients younger than 40 years.

Based on prehospital care data, patients were already hypotensive and tachycardic at the trauma scene, and at admission to the ER they had signs of shock, acidosis, severe base deficit, and increased arterial lactate. The greater need for endotracheal intubation in prehospital care among patients who did not survive may represent its greatest severity. The sequential measurements of base deficit and arterial lactate are considered reliable parameters to estimate the severity of hemorrhagic shock^24^, in agreement with our identification of the base deficit before EPP as a predictive factor of mortality.

To date, the association between the type of pelvic fracture and the urgent need for hemostasis is still controversial^8^. Our study proves that even fractures considered of minor severity can cause arterial injury, reinforcing the current consensus that the hemodynamic condition should determine the treatment strategy, rather than the type of fracture^8^.

High-energy trauma mechanisms can result in multiple injuries. More than 90% of patients with pelvic fractures have lesions in other body segments^25^. The prevalence of severe extrapelvic injuries that may justify shock is reported in up to 50% of patients^4^. However, in our study, this condition was even more prevalent (76.47%), making it difficult to prioritize treatment and, possibly, increasing hemostasis time.

Other studies that published their experience with EPP^5^ ^-^ ^7^ ^,^ ^9^ ^,^ ^10^ ^,^ ^12^ ^-^ ^16^ ^,^ ^21^ ^,^ ^26^ ^-^ ^29^ also reported a high mean ISS, between 30 and 55, though with lower mortality, ranging from 7.14% to 36.3%. Cheng et al.^6^ reported 61% of patients with extrapelvic lesions with AIS ≥4, while Burlew et al.^5^ reported that 85% of patients required other surgical procedures (on average, three procedures in addition to EPP and pelvis EF), with a mean ISS of 48. Associated mild injuries can result in a synergistic effect on mortality when combined with pelvic fractures^6^, especially in patients with traumatic brain injury and shock. The presence of an associated abdominal lesion requiring exploratory laparotomy is reported between 50% and 77.77% in the series that published the EPP results^9^ ^,^ ^14^ ^,^ ^28^ ^,^ ^30^.

In the present study, in addition to the absence of significant difference in the time between hospital admission and EPP between the groups analyzed, this time can be considered long for both. Only Totterman et al.^14^ reported a similar time between hospital admission and surgery (134 [5-720] minutes), with a mortality of 28%. In other studies^5^ ^-^ ^7^ ^,^ ^9^ ^,^ ^10^ ^,^ ^12^ ^,^ ^13^ ^,^ ^16^ ^,^ ^21^ ^,^ ^26^ ^-^ ^28^ the mean time to EPP ranged from 22 to 82 minutes, also resulting in lower mortality among packed patients, from 7.14% to 36.3%. Clarke et al.^31^ analyzed patients with blunt abdominal trauma and hemodynamic instability and showed that mortality increased by 1% for every three minute delay in achieving bleeding control.

Several studies^5^ ^,^ ^7^ ^,^ ^9^ ^,^ ^10^ ^,^ ^14^ ^,^ ^16^ ^,^ ^29^ have shown a significant improvement in hemodynamic parameters and a decrease in the need for blood transfusions after EPP^5^ ^,^ ^9^ ^,^ ^10^ ^,^ ^16^ ^,^ ^21^ ^,^ ^26^ ^,^ ^28^ ^,^ ^29^. However, although the progressive improvement in MAP, pH, and base deficit values represent evidence of improvement in hemodynamic status, this effect cannot be attributed exclusively to EPP, as the patients underwent other surgical procedures and received blood transfusions and vasopressors.

On the other hand, arterial lactate had a significant progressive increase in both groups. Abramson et al.^32^ reported the relationship between normalization of lactate values and mortality in trauma patients. In their study, all patients whose lactate normalized within 24 hours survived, while the survival rate decreased to 77.8% and 13.6%, respectively, when lactate took 48 hours or more to return to normal values.

Hemostatic resuscitation should be started early, as 54% to 80% of hypotensive patients do not respond to initial resuscitation^4^. Considering studies that published their experiences with EPP^5,7,9,10,12 14,21,26,28,29^, all but the one published by Cheng et al.^6^ found a higher mean number of units transfused before EPP, varying from 3.7 to 12 PRBC units. In addition, these transfusions took less time, ranging from 22 (±8) minutes to 82 (±13) minutes, except in the study published by Totterman et al.^14^. The greater amount of PRBC received in less time before EPP likely resulted in improved tissue perfusion and bleeding control at an earlier stage, before the development of coagulopathy. In addition, the need for transfusions after EPP and in the next 24 hours decreased^5^ ^,^ ^9^ ^,^ ^10^ ^,^ ^12^ ^,^ ^21^ ^,^ ^26^ ^,^ ^28^ ^,^ ^29^. In our series, patients who died received significantly more blood products. Transfusions of PRBC, FFP, and platelets increased in the 24 hours after EPP, probably due to the lower amount of blood products received before EPP, and to the higher incidence of coagulopathy, which we observed in 70.58% of patients in the first 12 hours of hospitalization, higher than previously reported in the literature, from 25% to 40%^33^. Coagulopathy is the main cause of failure to control pelvic bleeding, both by EPP and by angioembolization^4^, and it is an independent predictor of mortality in patients with pelvic fractures^23^. Gaski et al.^34^ reported that the institution of a massive transfusion protocol with a higher amount of FFP and platelets, even with a lower amount of transfused PRBC, resulted in a reduced need of EPP, without a significant change in the mortality rate.

Patients with refractory shock after resuscitation with adequate volume and mechanical stabilization of the pelvis are more likely to have arterial injury^35^, with a reported incidence greater than 50%^7^ ^,^ ^14^ ^,^ ^36^ ^-^ ^40^, making this the most frequent indication for arteriography^41^ ^,^ ^42^. In studies on EPP results^5^ ^-^ ^7^ ^,^ ^9^ ^,^ ^10^ ^,^ ^12^ ^-^ ^16^ ^,^ ^21^ ^,^ ^26^ ^-^ ^29^, arteriography was performed in a complementary way in 13,33% to 100% of patients, and the presence of arterial injury was confirmed in 33.33% to 100% of cases. The prevalence of arterial injury in these studies varied widely, from 14.25% to 88% among patients with hemodynamic instability undergoing EPP. Therefore, although venous injuries are thought to be responsible for retroperitoneal bleeding in 80% to 90% of patients with pelvic fractures, when considering patients with persistent hemodynamic instability after resuscitation with volume, external fixation, and EPP, the presence of arterial injury should be excluded by arteriography. In developing countries such as Brazil, this resource is often not available, especially in public hospitals, where most trauma patients are hospitalized. EPP does not prevent arteriography and may even reduce its need^5^. In our series, arteriography was performed in less than half of patients with indication, considering the recommendations of current guidelines^8^ ^,^ ^43^ ^-^ ^45^. According to Tesoriero et al.^46^, up to 80% of deaths in patients with pelvic fractures can be attributed to lack of bleeding control and delays in hemostatic procedures.

Mortality of patients with pelvic fracture and hemodynamic instability is reported to range from 21% to 66%^2^ ^,^ ^4^ ^,^ ^6^ ^,^ ^7^ ^,^ ^9^ ^,^ ^11^ ^-^ ^13^ ^,^ ^16^ ^,^ ^21^ ^,^ ^29^ ^,^ ^47^. Among these, the lowest mortality rates are found among patients undergoing EPP5^7,9,10,12 16,27 29^, ranging from 7.14% to 36.3%. The mortality rate of 56.86% observed in this study is consistent with that reported in the literature. However, this value is higher than the other publications that assigned EPP a priority role in the treatment of pelvic bleeding.

This study has several limitations, including its observational and retrospective nature. Furthermore, we included only patients undergoing EPP, preventing comparison of treatment results with other methods. As most patients had multiple associated serious injuries, the contribution of pelvic bleeding to the outcome should be interpreted with caution. Information on the volume of crystalloid received during prehospital care and at admission to the emergency department was not clearly recorded in many of the patients’ charts, representing a potential bias that could influence the incidence of coagulopathy and mortality. In addition, recent recommendations suggest earlier use of blood products and fewer crystalloids during resuscitation and, as we included patients from 2010 to 2016, changes in the resuscitation of these patients may have impacted results. However, although the number of patients analyzed can be considered small, only three studies^5^ ^,^ ^16^ ^,^ ^29^ reported the results of EPP in a larger population, in trauma centers inserted in trauma systems, with conditions different from those of hospitals in developing countries, with fewer resources.

## CONCLUSION

Age and pre-EPP base deficit are independent predictors of mortality in patients with pelvic fracture and hemodynamic instability. Therefore, older patients and those with a greater base deficit before EPP should be recognized as more severe. These patients should be prioritized for rapid control of pelvic bleeding with extraperitoneal pelvis packing, adequate fluid resuscitation, external pelvic fixation, and complementary angioembolization when indicated.
